# Treatment of hyperprolactinaemia reduces total cholesterol and LDL in patients with prolactinomas

**DOI:** 10.1007/s11011-016-9882-2

**Published:** 2016-08-15

**Authors:** Verena Schwetz, Rosaria Librizzi, Christian Trummer, Georg Theiler, Claudia Stiegler, Thomas R. Pieber, Barbara Obermayer-Pietsch, Stefan Pilz

**Affiliations:** 1 Division of Endocrinology and Metabolism, Department of Internal Medicine, Medical University of Graz, Auenbruggerplatz 15, 8036 Graz, Austria; 2 Department of Internal Medicine, Feldbach Regional Hospital, Feldbach, Austria

**Keywords:** Hyperprolactinaemia, Prolactinoma, Lipid metabolism, Total cholesterol, LDL

## Abstract

Previous studies suggest that hyperprolactinaemia might have adverse effects on lipid and glucose metabolism. We therefore aimed to evaluate whether dopamine agonist treatment with cabergoline has significant effects on blood lipids, fasting glucose and HbA1c levels in patients with micro- or macroprolactinoma. In this retrospective observational study the main outcome measures are changes in parameters of glucose and lipid metabolism compared at hyperprolactinaemia and after achievement of normoprolactinaemia by cabergoline treatment. We enrolled 53 study participants (22 females; median [interquartile range] age: 40.0 [27.5 to 50.0] years), 22 (41.5 %) with micro-, and 31 (58.5 %) with macroprolactinomas. After a median follow-up of 9 months, prolactin levels decreased from 220.6 (80.7–913.4) to 11.2 (3.5–18.7) ng/mL (*p* < 0.001). There was a significant decrease in median levels of low-density lipoprotein (LDL) from 121.6 (±39.4) to 110.6 mg/dl (±37.6, *p* = 0.005) and total cholesterol from 191 (168.5–241) to 181 mg/dl (162–217, *p* < 0.001), but no change in high-density lipoprotein (HDL), triglycerides, fasting glucose and HbA1c. We observed a significant increase in testosterone in men and in oestradiol in women. In linear regression analyses using the change in total cholesterol or LDL as dependent, and the change in prolactin, oestradiol, and testosterone as independent variables, no significant predictor of the change in total cholesterol or LDL was identified. In patients with prolactinomas, normalisation of elevated prolactin levels by cabergoline treatment was accompanied by significant reductions in LDL and total cholesterol. Further studies are warranted to confirm our findings and to evaluate the clinical implications of lipid levels in the monitoring and treatment of patients with prolactinomas.

## Introduction

Prolactinomas are the most common hormone-secreting pituitary tumours. In addition to the known classical symptoms of hyperprolactinaemia, elevated levels of prolactin due to micro- or macroprolactinoma could have negative influences on metabolic parameters.

Compared to controls or patients with adequately treated hyperprolactinaemia, patients with hyperprolactinaemia have an adverse metabolic profile with higher levels of triglycerides, HOMA-IR (homeostatic model assessment – insulin resistance), lower adiponectin (de Assuncao Alves Rodrigues et al. 2012) and impaired glucose tolerance (Tourniaire et al. 1974), suggesting a diabetogenic effect of prolactin (Landgraf et al. 1977). It is, however, still largely unknown whether treatment of hyperprolactinaemia has significant effects on glucose and lipid metabolism. Existing knowledge on this topic is based on small retrospective studies including 21 to 61 patients treated by the dopamine agonist cabergoline in order to normalize their elevated prolactin levels. These studies revealed inconsistent results regarding the changes in parameters of lipid and glucose metabolism that were observed before and after cabergoline treatment (Ciresi et al. 2013; Inancli et al. 2013; dos Santos Silva et al. 2011; Auriemma 2013). In detail, 6 to 12 months of cabergoline treatment were followed by a reduction in low-density lipoprotein (LDL), cholesterol (Ciresi et al. 2013; Inancli et al. 2013; dos Santos Silva et al., 2011), total cholesterol (Ciresi et al. 2013), triglyceride levels (Ciresi et al. 2013; dos Santos Silva et al. 2011), an increase in high-density lipoprotein (HDL) (Ciresi et al. 2013), a decrease in HOMA-IR (Ciresi et al. 2013; dos Santos Silva et al. 2011), and an increase in measures of insulin sensitivity (Ciresi et al. 2013; Inancli et al. 2013). One study showed a decrease in HbA1c (Ciresi et al. 2013), another study in fasting glucose levels (dos Santos Silva et al. 2011). Additionally, the study by Inancli et al. (2013) found an improvement in inflammatory markers and a decrease in carotid-intima-media-thickness independent from the decrease in prolactin, LDL, cholesterol and body mass index (BMI) (Inancli et al. 2013).

Regarding body composition, an observational study showed that compared to controls and patients with normoprolactinaemia under cabergoline treatment, newly-diagnosed men with prolactinomas had higher body fat content (Naliato et al. 2008). Other studies, however, showed no difference in body fat in women with prolactinoma compared to controls (Naliato et al. 2007) and did not find a change in BMI after 6 months of cabergoline treatment. By contrast, Ciresi et al. (2013) found a reduction in waist circumference after 12 months of cabergoline treatment.

From a pathophysiological point of view some effects of prolactin exist that may underlie the association between prolactin and metabolic parameters such as the direct effect of prolactin on adipose tissue and on the lipoprotein lipase (LPL). Furthermore, the change in sex hormones might alter the lipid profile and dopamine agonist therapy per se might have an influence on metabolic parameters.

The aim of this study was to evaluate parameters of glucose and lipid metabolism in patients with micro- or macroprolactinoma before initiation of treatment and after achievement of normoprolactinaemia. We hypothesized that glucose and lipid parameters might improve after restoration of normoprolactinaemia.

## Subjects and methods

### Subjects

We carried out a retrospective chart review of patients with hyperprolactinaemia who presented at the outpatient clinic of the Division of Endocrinology and Metabolism, Department of Internal Medicine, Medical University of Graz, Austria, between April 2004 and March 2014. Patients with secondary hyperprolactinaemia due to drugs (including neuroleptics, antidepressants, opiates and gastrointestinal prokinetics) or patients with mixed-secreting tumours, patients already on dopamine agonists at their baseline visit and those patients who didn’t show normoprolactinaemia at their follow-up visit were excluded from analysis.

Patients receiving oestrogens and/or progesterone as contraceptives, postmenopausal women on hormone replacement therapy and patients on anti-hyperglycaemic agents or on medications influencing lipid metabolism (statins, fibrates) were further excluded. Additionally, we did not include patients with multiple pituitary hormone deficiencies and patients with hypogonadism due to causes other than prolactinoma itself. The Ethics Committee of the Medical University of Graz approved this study.

### Procedures

Standard anthropometric data (height, weight, blood pressure) were obtained from each subject at each routine visit and documented in the medical charts. The BMI was calculated as the weight in kilograms divided by the square of height in meters. Basal blood samples for hormonal (prolactin, total testosterone (TT), free testosterone (FT), sex-hormone binding globulin (SHBG), free triiodothyronine (fT3), free thyroxine (fT4), thyroid-stimulating hormone (TSH), cortisol, adrenocorticotropic hormone (ACTH), human growth hormone (HGH), insulin-like growth factor 1 (IGF-1) and metabolic parameters (glucose, insulin, total cholesterol, LDL, HDL, triglycerides) were collected between 8.00 and 9.00 a.m. after an overnight fast. All hormones were measured on a daily basis and stored at 4° until analysis.

Parameters in the state of hyperprolactinaemia were compared with parameters after achievement of normoprolactinaemia, regardless of the time span.

### Biochemical analyses

Prolactin, total testosterone, TSH, fT3, fT4, cortisol, ACTH, HGH and IGF-1 were measured by luminescence immunoassay (Siemens, Erlangen, Germany). Insulin was measured by ELISA (Siemens, Erlangen, Germany). Fasting glucose, HbA1c, triglycerides, total cholesterol, HDL cholesterol, and LDL cholesterol were measured using Modular Analytics SWA (Roche, Basel, Switzerland). Coefficients of variation for all parameters analyzed were <10 %.

### Statistical analyses

Data are presented as mean and standard deviation (SD) when both variables (i.e. before and after) were normally distributed, when both or one variable was non-normally distributed median and interquartile ranges (IQR) were used. Kolmogorov-Smirnov test and descriptive statistics were used to evaluate the distribution of data. All continuous parameters following a non-normal distribution were logarithmically transformed when parametric tests were performed.

Pearson correlation analysis was used to test for associations between prolactin and baseline laboratory parameters. To compare parameters of glucose and lipid metabolism before and after cabergoline treatment, the Student’s paired T-test was used. Multivariable stepwise linear regression analysis was performed with the change in total cholesterol and the change in LDL as dependent variables and the change in prolactin, oestradiol, testosterone as independent variables. Men and women were analysed separately to account for the different reference ranges of oestradiol and testosterone in males and females. In addition, we performed sex-stratified analyses to study effects of cabergoline treatment on sex hormones in both genders. A *p*-value of 0.05 was considered statistically significant. All analyses were performed using SPSS 21.0 (SPSS Inc., Chicago, IL).

## Results

We screened 450 patients of whom 53 were included in the present analysis based on the availability of a prolactinoma diagnosis and the availability of metabolic parameters before and after cabergoline treatment. We enrolled 53 patients with either micro- or macroprolactinomas (22 females, 31 males) with a median age of 40 (27.5–50.0) years with newly diagnosed micro- or macroprolactinoma. According to the prolactinoma guidelines of the Endocrine Society, the diagnosis was based on clinical signs of hyperprolactinaemia, elevated levels of prolactin and a pituitary tumour on MRI (Melmed et al. 2011). Twenty-two patients (41.5 %) had micro-, 31 (58.5 %) had macroprolactinoma. Thirty-eight patients had hypogonadism (71.7 %), 11 did not (20.8 %), 2 were on hormone therapy and could thus not be evaluated, for 2 patients data were insufficient to determine whether hypogonadism was present. In macroprolactinoma patients, 23 patients had hypogonadism, 1 had hypogonadism and secondary hypothyroidism. After normalization of prolactin levels, hypogonadism persisted in 18 patients.

Laboratory and anthropometric parameters are shown in Table 1. There was no significant difference in metabolic parameters between patients with micro- and macroprolactinomas (data not shown). At baseline, 1 patient had impaired fasting glucose (defined as fasting glucose from 110 to 125 mg/dl), 1 patient had a fasting glucose ≥126 mg/dl, i.e. diabetes mellitus. At follow-up, again 1 patient had impaired fasting glucose, but 2 patients had fasting glucose ≥126 mg/dl. At baseline, there was a significant correlation between prolactin and triglycerides (β = 0.294, *p* = 0.033), glucose (β = 0.312, *p* = 0.025) and total testosterone (β = 0.363, *p* = 0.008), but not HbA1c or levels of total cholesterol, LDL and HDL. There was no significant difference in thyroid hormone levels before and after normalization of prolactin levels (see Table 1).

**Table 1:** Patient characteristics and laboratory parameters at baseline and after achievement of normoprolactinaemia. Data are presented as mean and standard deviation (SD) when both variables (i.e. before and after) were normally distributed (i.e. BMI, LDL, Hb), when both or one variable was non-normally distributed median with interquartile ranges (IQR) was used. Student’s T-test was used to compare data at baseline and follow-up (after transformation of non-normally distributed variables). The *p*-values are listed in the right column. A p-value of <0.05 was considered statistically significant

	Baseline		Follow-up		p-value
*n* = 53 22 women, 31 men	Median or mean	IQR or SD	Median or mean	IQR or SD	
Age (years)	40	27.5–50			
BMI (kg/m^2^)	27.9	5.9	28.6	5.6	0.396
Prolactin (ng/ml)	220.6	80.7–913.4	11.2	3.5–18.7	<0.001
Total cholesterol (mg/dl)	191	168.5–241	181	162–217	<0.001
LDL (mg/dl)	121.6	39.4	110.6	37.6	0.005
HDL (mg/dl)	54	43–69	53	44–70	0.068
Triglycerides (mg/dl)	91	62–155	98	64–131	0.173
Fasting glucose (mg/dl)	88	81–93	86	82–94	0.445
HbA1c (mmol/mol)	37	35–38	34	32–39	0.727
Oestradiol (women only) (pg/ml)	20.0	13.1–29.8	45.1	21.1–93.1	0.001
Total testosterone (men only) (ng/ml)	1.83	1.45–2.59	2.27	1.78–4.29	0.008
LH (mIE/ml)	1.7	0.7–3.1	2.1	0.6–3.9	0.388
FSH (mIE/ml)	4.2	2.4–6.9	4.8	2.6–8.9	0.168
TSH (μU/ml)	1.85	1.32–2.41	1.84	1.28–2.60	0.768
fT4 (pmol/L)	13.9	12.5–15.3	13.3	12.5–14.6	0.323
fT3 (pmol/L)	4.8	4.4–5.2	4.9	4.5–5.4	0.050

BMI = body mass index

LDL = low-density lipoprotein

HDL = high-density lipoprotein

HbA1c = glycated haemoglobin

LH = luteinizing hormone

FSH = follicle-stimulating hormone

Hb = haemoglobin

TSH = thyroid-stimulating hormone

fT4 = free thyroxine

fT3 = free triiodothyronine

LDL = low-density lipoprotein. LDL in mg/dl

**Fig. 1:**
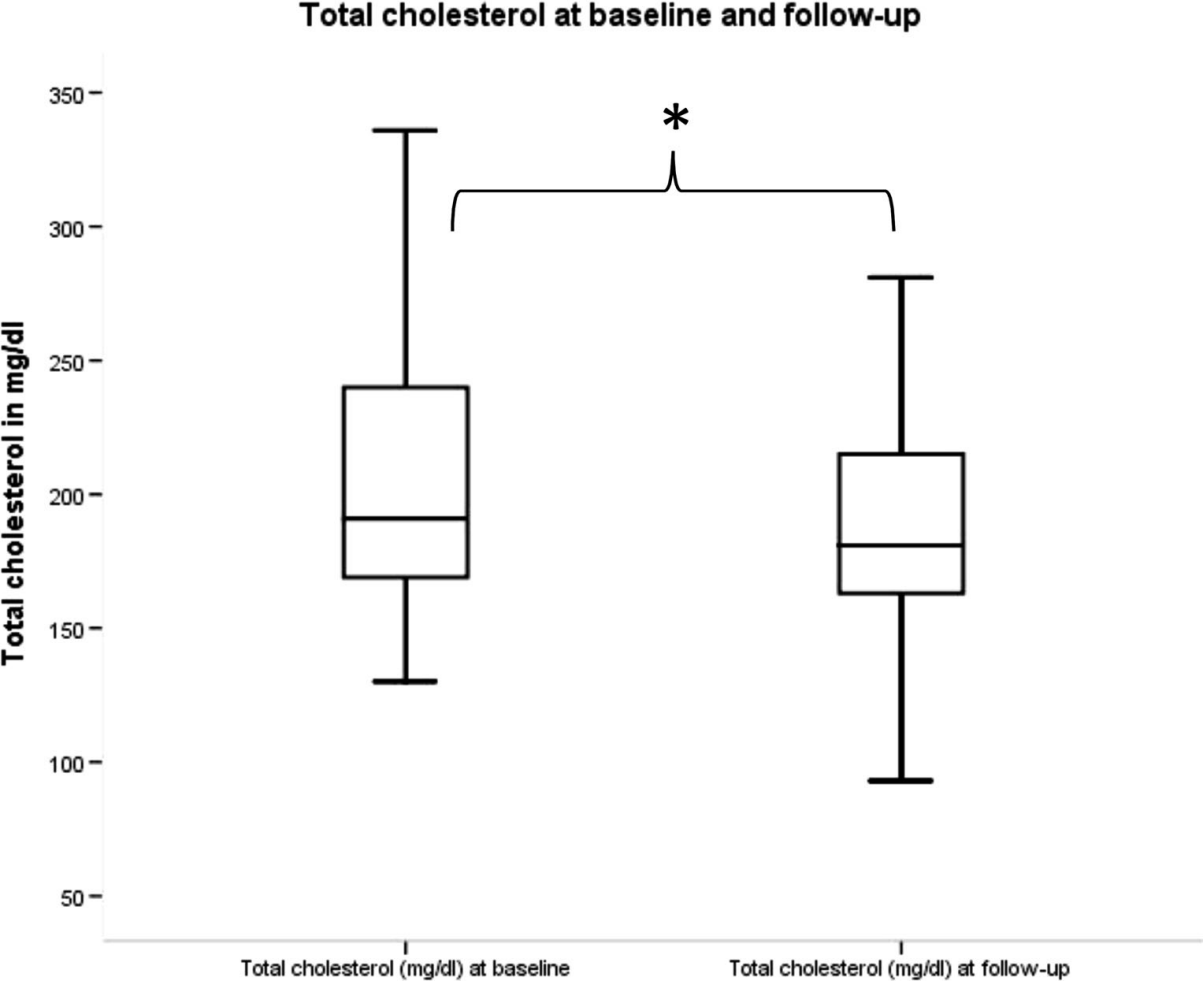
Change in total cholesterol between baseline and follow-up. * implies a significant change. Total cholesterol at baseline and at follow-up. Total cholesterol in mg/dl

**Fig. 2:**
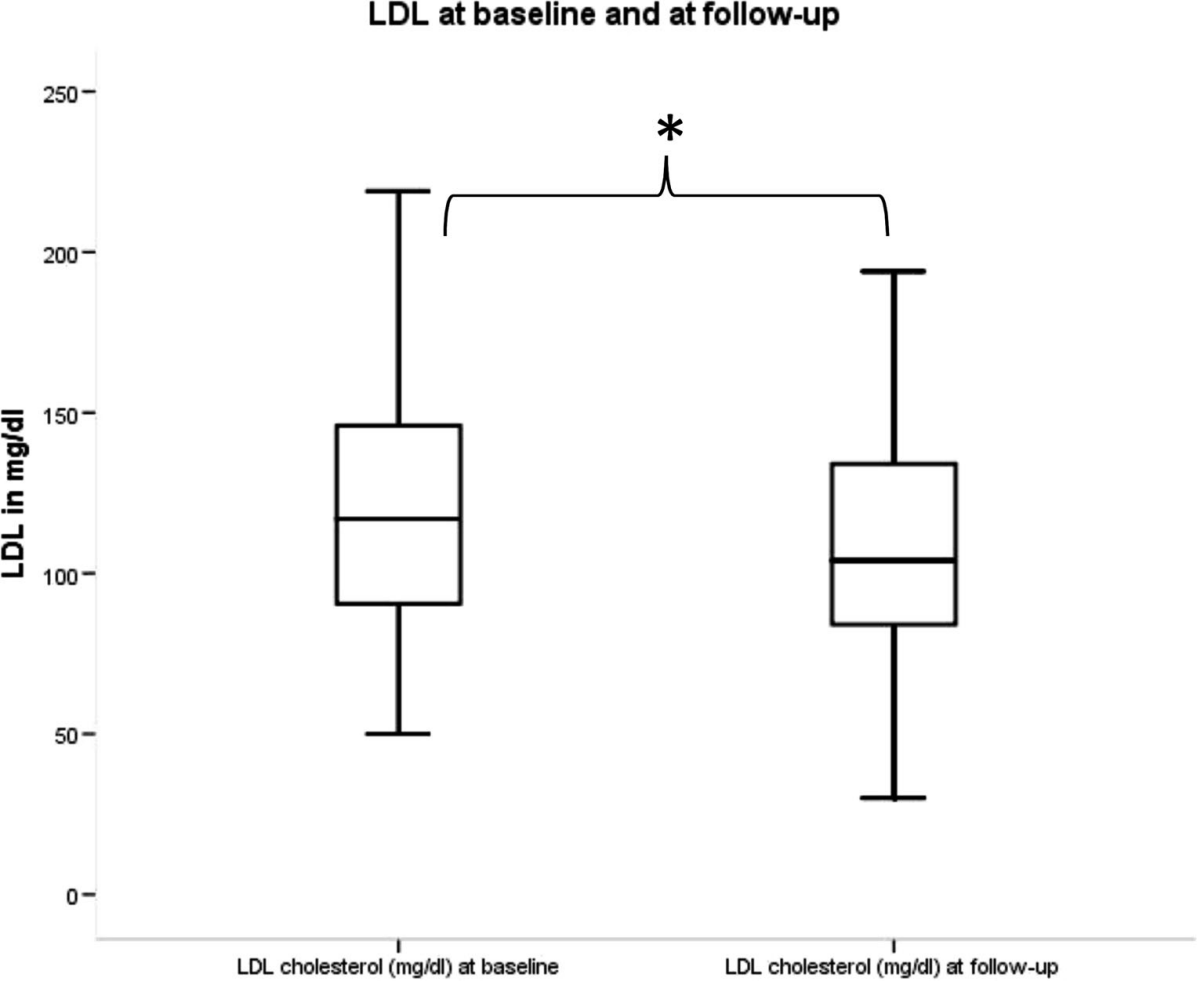
Change in LDL between baseline and follow-up. * implies a significant change. LDL at baseline and at follow-up

After treatment with cabergoline at a median dose of 0.5 mg (0.5–0.9) per week, patients were re-evaluated and found to have normoprolactinaemia after a median time of 9 months (IQR 4–16). There was a significant decrease in levels of total cholesterol (Fig. 1) and LDL (Fig. 2), but no significant difference in levels of HDL, triglycerides, fasting glucose, HbA1c. Testosterone did not, while oestradiol did show a significant increase (Table 1) in all patients analysed together. When analysing all 31 men separately, there was a significant increase in testosterone, analysing the 22 women separately, there was a significant increase in oestradiol. In our patient cohort, we did not observe any significant correlation between cabergoline dose and the change in total cholesterol and LDL cholesterol.

In linear regression analyses, analysing men and women separately, using the change in total cholesterol or LDL as dependent variable, and the change in prolactin, oestradiol (for women), and testosterone (for men) as independent variables, no significant predictor of the change in total cholesterol and LDL could be determined (data not shown). In linear regression analyses using the same dependent and independent variables, analyzing patients with micro- and macroprolactinomas separately, again no significant predictor of the change in total cholesterol and LDL could be determined (data not shown).

## Discussion

In a retrospective analysis of 53 patients with micro- or macroprolactinoma, we observed that restoration of normoprolactinaemia by cabergoline treatment led to a significant decrease in total cholesterol and LDL levels but there was no change in parameters of glucose metabolism.

Our findings significantly add to the existing literature as we analysed, to the best of our knowledge, one of the largest study cohorts with prolactinomas with respect to parameters of lipid and glucose metabolism before and after cabergoline treatment. Our results are in line with most published studies to date in terms of lipid metabolism, but we did not confirm previously described changes in glucose metabolism after cabergoline treatment. Underlying reasons for these inconsistent results remain speculative but may be due to different study characteristics and the risk of bias in uncontrolled studies. Furthermore, the lack of significant change in glucose levels in our study may be related to the fact that the majority of the patients investigated had normal glucose levels to start with. Nevertheless, our results strongly support the notion that treatment of hyperprolactinaemia may have beneficial effects on lipid metabolism. This is well in line with cross-sectional studies showing associations between high prolactin and adverse lipid profiles (Ciresi et al. 2013; Inancli et al. 2013; dos Santos Silva et al. 2011) and in line with data published by Auriemma et al. (2013). After a follow-up of 12 months, at which 90 % of their patients had achieved normoprolactinaemia, only fasting insulin, HOMA-IR and total cholesterol had significantly declined compared to baseline; only a trend was observed for LDL. After 60 months a significant improvement was reported in all metabolic parameters, including LDL, TG, HDL, weight, BMI and visceral adiposity index. These data, together with our results presented here, might suggest that total cholesterol and LDL are the first metabolic parameters affected by a decrease in prolactin in patients with prolactinoma after initiation of cabergoline treatment. This effect seems to be independent of the cabergoline dose used. Continued treatment with cabergoline could imply normalization of all metabolic parameters.

The pathophysiological mechanism behind the associations of prolactin and lipids is less clear, however. Possible mechanisms include the direct effect of prolactin on adipose tissue, hypogonadism, and the role of dopamine. The retrospective design, however, does not allow drawing any definitive conclusions on the mechanisms underlying the changes observed.

Evidence in support of the first mechanism includes the detection of the expression of four prolactin receptor isoforms in human abdominal adipose tissue, implying a direct regulation of human adipose tissue by prolactin (Ling et al. 2003). Prolactin was also shown to inhibit LPL activity in human adipose tissue, a physiologic mechanism to regulate adipose tissue metabolism during lactation (Ling et al. 2003). LPL is the essential and rate-limiting enzyme for the hydrolysis of the triglyceride core of circulating triglyceride-rich lipoproteins, chylomicrons, and very low-density lipoproteins (VLDL) to provide free fatty acids (Wang and Eckel 2009). These free fatty acids are needed for the enhanced lipid metabolism, i.e. production of triglycerides from fatty acids, in the mammary gland during lactation, where lipid production in the adipose tissue is decreased (Flint et al. 2003; Ben-Jonathan et al. 2006). Another potential mechanism possibly contributing to the dysmetabolic changes, weight gain and redistribution of fat in patients with hyperprolactinaemia could be hypogonadism, as is known from data of women in menopause (Tchernof et al. 2000). Hyperprolactinaemia frequently causes hypogonadism which could contribute to the observed adverse metabolic profile in these patients. To account for this fact, we performed linear regression analysis to determine whether the change in testosterone and oestradiol levels predicts the change in total cholesterol and LDL. However, no significant association was found which might imply that the change in lipid profile is not mainly caused by hypogonadism as suggested by other authors (Ciresi et al. 2013).

The third potential mechanism is the role of dopamine in increasing energy expenditure and decreasing food intake (Ben-Jonathan and Hnasko 2001). In conditions of hyperprolactinaemia, levels of dopamine are lower, which in turn may negatively affect body weight and fat content. The administration of dopamine agonists in rodents led to a reduction in the retroperitoneal fat pad weight, an improvement in glucose tolerance, and a decrease in nocturnal lipolysis (Cincotta and Meier 1995). In type 2 diabetics, bromocriptine reduced fasting and post-prandial glucose, fasting and post-prandial triglycerides as well as fasting and post-prandial free fatty acids (Cincotta et al. 1999). In 9 hyperprolactinaemic women on bromocriptine therapy, total cholesterol and LDL showed a significant decrease (Fahy et al. 1999). Furthermore, patients with normal prolactin levels under dopamine agonist treatment (64.5 % with bromocriptine and 35.5 % with cabergoline) have lower body fat content than those with elevated prolactin levels, possibly due to metabolic effects of dopamine receptor 2 activation (Naliato et al. 2007). Similar findings were observed in patients receiving cabergoline, where only men showed a decrease in BMI and fat percentage after cabergoline treatment. Men as well as women in this study, however, showed a significant decrease in LDL levels, which is in line with our findings (Berinder et al. 2011).

We have to acknowledge that our data are limited as they are derived from a retrospective observational study and final conclusions regarding causality cannot be drawn from such study designs. Further limitations are the relatively low number of participants and the short follow-up, the latter partly owing to the aim of comparing baseline data of glucose and lipid parameters to data at first restoration of normoprolactinaemia under cabergoline. The main strengths are, however, that this is the second largest cohort focusing on this topic and that we could reproduce established knowledge by showing a significant impact of cabergoline treatment on prolactin, oestradiol and testosterone levels, which underlines the validity of our investigation. Further, we included only patients who achieved restoration of normoprolactinaemia in the period of time observed, in contrast to the study by Auriemma et al. (2013), where 7–10 % (depending on the time of follow-up) of the patients remained on elevated prolactin levels.

In conclusion, we observed a decrease in total cholesterol and LDL after restoration of normoprolactinaemia in 53 patients with prolactinoma. Considering the relatively high prevalence of hyperprolactinaemia and the clinical significance of lipid metabolism for clinical outcomes we recommend further studies to confirm our findings and elucidate the underlying pathophysiological mechanisms for the suggested link between prolactin and lipid metabolism. From a clinical point of view, our findings support considerations to monitor lipid metabolism in patients with prolactinoma as they are at an increased metabolic risk that may be ameliorated by restoration of normoprolactinaemia.
